# Adaptive Centipede Walking via Synergetic Coupling Between Decentralized Control and Flexible Body Dynamics

**DOI:** 10.3389/frobt.2022.797566

**Published:** 2022-04-05

**Authors:** Kotaro Yasui, Shunsuke Takano, Takeshi Kano, Akio Ishiguro

**Affiliations:** ^1^ Frontier Research Institute for Interdisciplinary Sciences, Tohoku University, Sendai, Japan; ^2^ Research Institute of Electrical Communication, Tohoku University, Sendai, Japan; ^3^ Graduate School of Engineering, Tohoku University, Sendai, Japan

**Keywords:** multi-legged locomotion, centipede, decentralized control, sensory feedback, irregular terrain, self-organization, inter-limb coordination, flexible body dynamics

## Abstract

Multi-legged animals such as myriapods can locomote on unstructured rough terrain using their flexible bodies and legs. This highly adaptive locomotion emerges through the dynamic interactions between an animal’s nervous system, its flexible body, and the environment. Previous studies have primarily focused on either adaptive leg control or the passive compliance of the body parts and have shown how each enhanced adaptability to complex terrains in multi-legged locomotion. However, the essential mechanism considering both the adaptive locomotor circuits and bodily flexibility remains unclear. In this study, we focused on centipedes and aimed to understand the well-balanced coupling between the two abovementioned mechanisms for rough terrain walking by building a neuromechanical model based on behavioral findings. In the behavioral experiment, we observed a centipede walking when part of the terrain was temporarily removed and thereafter restored. We found that the ground contact sense of each leg was essential for generating rhythmic leg motions and also for establishing adaptive footfall patterns between adjacent legs. Based on this finding, we proposed decentralized control mechanisms using ground contact sense and implemented them into a physical centipede model with flexible bodies and legs. In the simulations, our model self-organized the typical gait on flat terrain and adaptive walking during gap crossing, which were similar to centipedes. Furthermore, we demonstrated that the locomotor performance deteriorated on rough terrain when adaptive leg control was removed or when the body was rigid, which indicates that both the adaptive leg control and the flexible body are essential for adaptive locomotion. Thus, our model is expected to capture the possible essential mechanisms underlying adaptive centipede walking and pave the way for designing multi-legged robots with high adaptability to irregular terrain.

## 1 Introduction

Many roboticists have developed walking robots inspired by multi-legged animals (Zhou and Bi, 2012; Buschmann et al., 2015; Aoi et al., 2017), because such animals can effectively move in complex environments using their multiple legs. Among various animals species with different numbers of legs, myriapods such as centipedes and millipedes have a characteristic body structure, that is, a flexible and an elongated body trunk and a large number of legs (Manton, 1965). These morphological features offer great advantages in terrestrial locomotion. Compared to the rigid and short-bodied animals (e.g., insects), the flexible and elongated bodies enable the animals to easily adapt their body posture to the landscape. This results in stable walking even on irregular terrains because the animal can secure sufficient ground contact points to support the body. Furthermore, a large number of legs realizes a walking performance robust to malfunction of some legs. Therefore, understanding the walking mechanisms of myriapods will contribute to designing multi-legged robots with high locomotor performance in harsh environments such as disaster areas.

As for myriapod walking on irregular terrains, although the inherent behavioral and neurobiological mechanisms are mostly unclear, researchers in bio-inspired robotics have explored the locomotor mechanisms using multi-legged robots and mathematical models. Such previous studies can be categorized into two main approaches. One approach aimed to reveal the role of passive body dynamics on locomotor performance using myriapod-like robots (Koh et al., 2010; Masuda and Ito, 2014; Kinugasa et al., 2017; Ozkan-Aydin et al., 2020; Ozkan-Aydin and Goldman, 2021). For instance, Ozkan-Aydin et al. (Ozkan-Aydin et al., 2020; Ozkan-Aydin and Goldman, 2021) developed a centipede-inspired robot with compliant joints at the body trunk and legs and systematically investigated the walking performance according to the gait patterns and flexibility of the body joints. However, all of these studies assumed predetermined and fixed gait patterns; therefore, the contribution of adaptive gait generation was not taken into account. The second approach has considered adaptive gait generation mechanisms for myriapod-like robot locomotion on irregular terrain (Matthey et al., 2008; Inagaki et al., 2010; Takahashi and Inagaki, 2016). Matthey et al. proposed a leg-and-body controller using chaotic oscillators and investigated its adaptability on uneven terrains in simulations (Matthey et al., 2008). Inagaki et al. proposed a decentralized and event-driven control scheme that realized the relay of ground contact points between neighboring legs, and successful walking over stair-like obstacles was demonstrated in simulations (Inagaki et al., 2010; Takahashi and Inagaki, 2016). However, the produced gaits in these studies were different from those of real myriapods, and thus the walking performance of the proposed controllers was not as good as that of multi-legged animals. Therefore, further investigation based on the biological understanding of the walking control mechanism is needed.

As suggested above, the passive dynamics of the flexible body and the adaptive gait control are both likely to be essential for understanding the performance of myriapod locomotion on irregular terrains. Accordingly, to overcome the limitation of the previous studies, we have employed a synthetic approach that combined behavioral experiments and mathematical modeling (Yasui et al., 2017a; Yasui et al., 2017b; Kano et al., 2017). Specifically, we constructed a simple physical model of the myriapod body and proposed hypothesized leg control mechanisms based on the behavioral findings. Although our model in these studies reproduced typical myriapod walking gaits on the flat terrain in simulation (Yasui et al., 2017a) and as a robot (Kano et al., 2017), the body was modeled with relatively rigid joints and adaptability to the irregular terrains was examined solely in the case of crossing a single gap along the ground.

In this study, we focused on a centipede (*Scolopendra subspinipes mutilans*) as a model animal and explored its adaptive walking control mechanisms using the synthetic approach, especially on irregular terrain. The centipede has a flexible body and uses a characteristic gait in which each leg on the ipsilateral side follows the ground contact point of its nearest anterior leg by propagating the wave of leg motions from the head to the tail (Yasui et al., 2017b). This gait seems effective for propulsion when the secure scaffolds are limited in unstructured environments. Accordingly, we observed the behavioral responses when a part of the terrain was suddenly removed and restored again during walking. As a result, we found that the legs losing footholds stopped periodic walking motions, and only restarted walking when the ground contact was restored. Based on this finding, we constructed a flexible-bodied model of the centipede, and proposed hypothesized control mechanisms that use local ground contact sense obtained at the legs and the ventral body surface, to generate adaptive walking. Using simulations, we validated our model could reproduce the adaptive centipede walking. Furthermore, we demonstrated the locomotor performance deteriorated on rough terrain when adaptive leg control was removed or when the body was rigid, which indicates that adaptive leg control and flexible body dynamics are both essential for high adaptability to irregular terrains.

## 2 Behavioral Experiments

**FIGURE 1:**
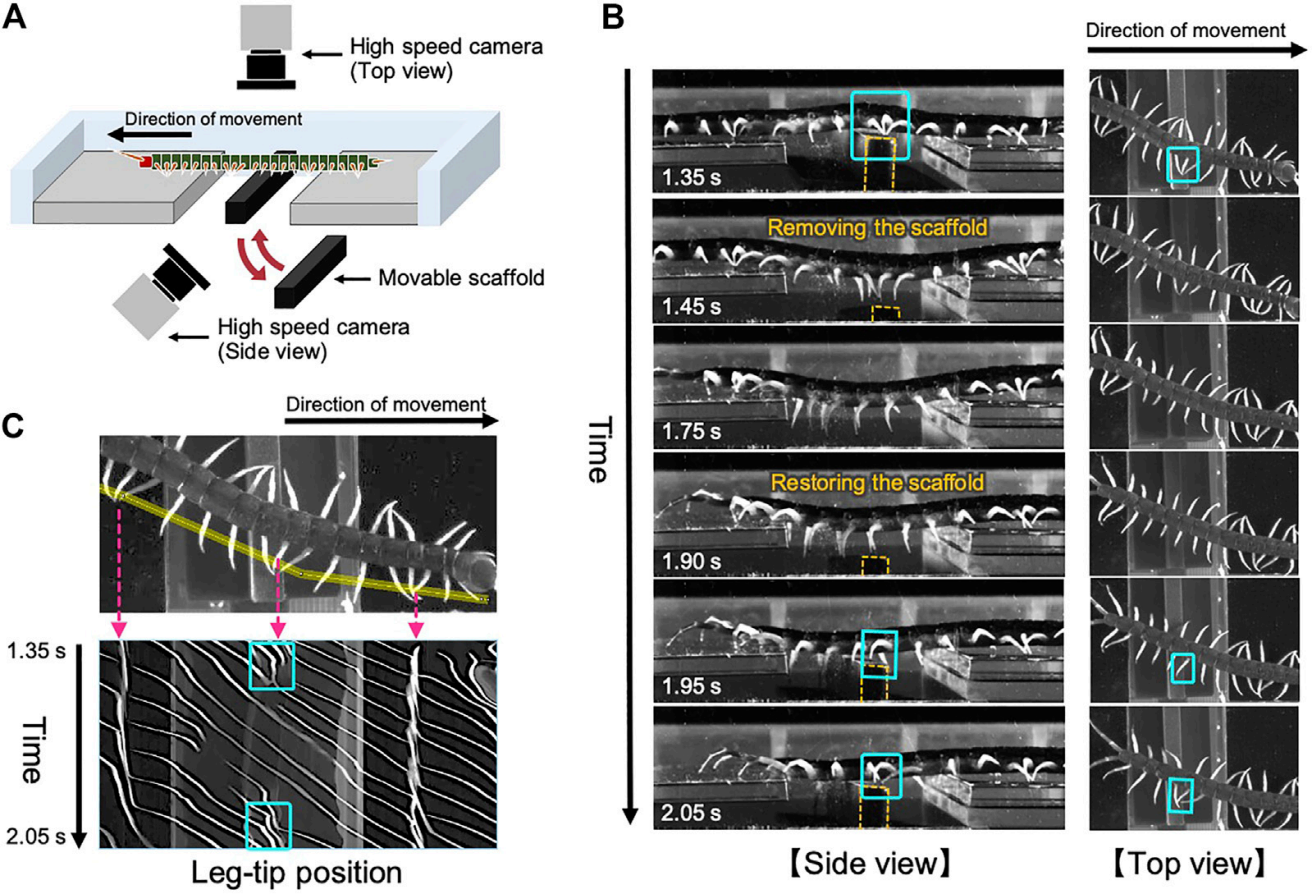
Adaptive centipede walking when part of the terrain was temporarily removed and subsequently restored during locomotion. **(A)** Schematic of the experimental setup. **(B)** Snapshots of the observed walking from the side and top views. All the snapshots are mirror images of their original ones. In the original movies (Supplementary movies S1 and S2), the direction of movement was from the right to the left. Orange dashed lines indicate the contour of the moving scaffold. **(C)** Spatiotemporal plots of the tip positions of the left legs along the body axis. Yellow line in the upper figure denotes the cropped line we used for the analysis. Cyan squares in **(B,C)** indicate the characteristic behavior observed on the moving scaffold that the posterior legs trail the ground contact point of the nearest anterior legs.

To investigate the adaptive locomotion of centipedes in response to the change in walking terrains, we performed behavioral experiments. Although we previously reported the behavioral changes when centipedes lose some footholds during walking (Yasui et al., 2017b), it remained unclear how they adapt their gait to the suddenly obtained scaffolds. Accordingly, here we observed the centipede walking when part of the terrain was temporarily removed and restored during locomotion (Figure 1A). More specifically, we prepared a rectangular solid as a movable scaffold and placed it at the gap area of the walking terrain at the beginning of the walking experiment. Then, we temporarily removed and restored the scaffold during walking by manual manipulation. Details of the experimental methods are described in the Materials and Methods section.

Snapshots of the observed adaptive walking (Supplementary Movies S1, S2) and spatiotemporal plots of the leg-tip positions are shown in Figures 1B,C. Behavioral findings from this experiment are summarized as follows:

(1) At the beginning of the experiment, the centipede initiated walking using the terrain between the gap areas as a scaffold. Specifically, legs in the posterior section exhibited footfall patterns trailing the ground contact points of their anterior legs, which was the characteristic behavior of the centipede using retrograde wave gait (Manton, 1965).
(2) When the terrain between the gap areas disappeared (1.45–1.75 s in Figure 1B), legs located over the gap gradually stopped rhythmic motion and paused in an extended position, whereas the other legs over the ground continued walking. This behavior was consistent with the previously reported results (Yasui et al., 2017b).
(3) Subsequently, when the removed terrain was restored between the gap areas (1.90–2.05 s in Figure 1B), legs contacting with the appeared terrain started the walking motion, and simultaneously the posterior legs began moving to trail the contact point of the anterior legs.

Most importantly, the abovementioned third behavioral finding was novel in this study and it suggests two control mechanisms for adaptive centipede walking. First, the local sensory information due to the suddenly obtained ground contact can initiate the walking motions of the legs that are in the resting state. Second, the posterior legs coordinate their motions to trail the contact point at the newly obtained scaffold in the anterior body section. These mechanisms are likely to be essential for reproducing the locomotor performance of centipedes adaptive to irregular terrains.

## 3 Model

In this study, we investigate the essential control mechanisms underlying adaptive centipede walking on irregular terrain with a synthetic approach using mathematical models. This section explains the mechanical model of the flexible-bodied centipede and proposes decentralized control rules for adaptive walking based on the behavioral findings.

### 3.1 Mechanical System

**FIGURE 2:**
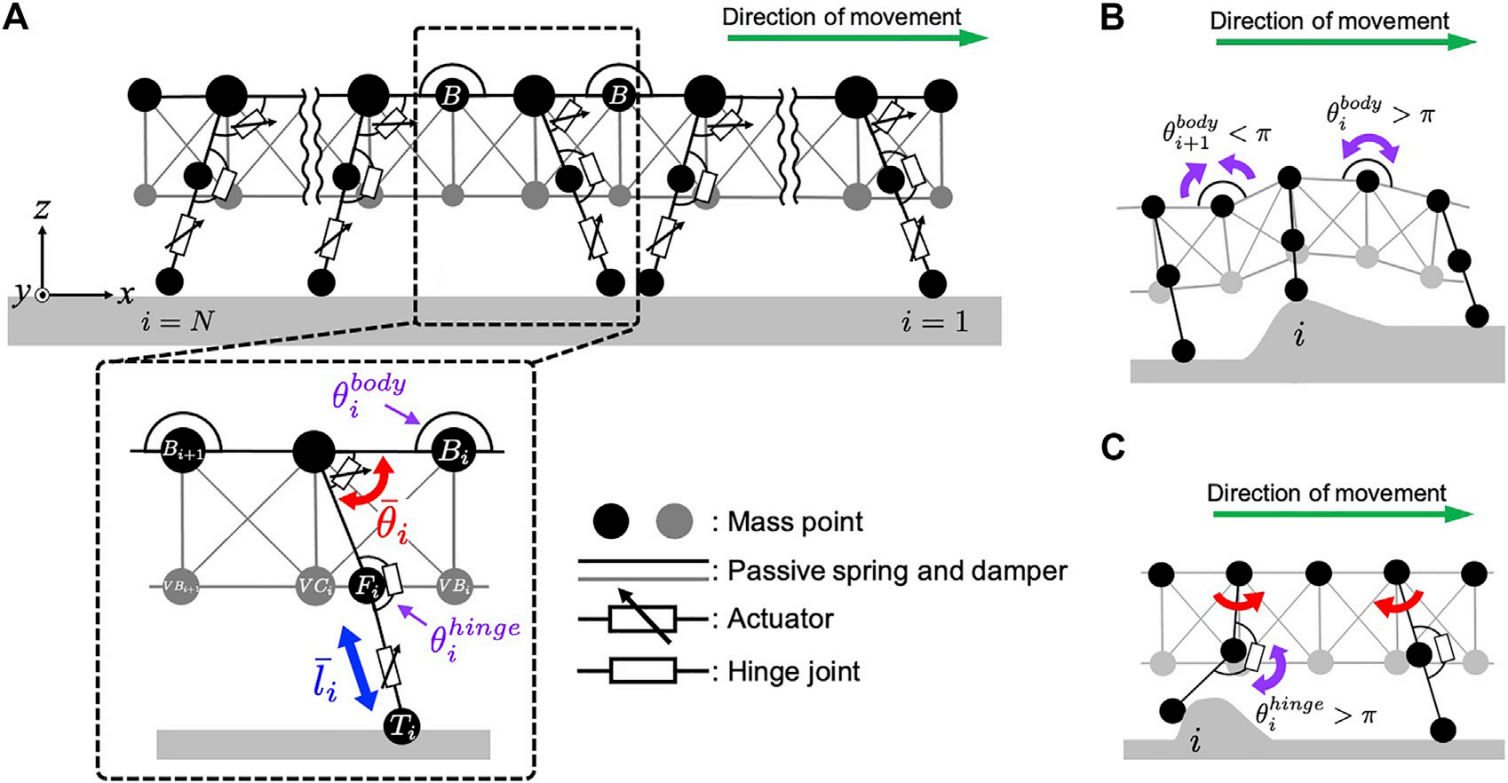
Schematic of the mechanical system in our centipede model. **(A)** Overview of the centipede body model that consists of the spring-mass-damper system. **(B)** The effect of the passive dynamics of the flexible body trunk. **(C)** The effect of the passive dynamics of the flexible leg joints.

For simplicity, we focused on the interlimb coordination at the ipsilateral side. Accordingly, we constructed a two-dimensional physical model of the centipede, wherein each body segment includes one leg. The centipede body was modeled based on a mass-spring-damper system (Figure 2A). To describe the flexible body trunk, mass points were connected in a mesh grid pattern *via* parallel combinations of a passive spring and a damper. Additionally, we implemented flexible passive torsional springs around the mass points located between the body segments at the dorsal side (refer to the mass points named “B” in Figure 2A), which made it easier to individually tune the flexibility of the back bending and the softness of the abdomen. Such flexibility allows the body trunk to passively bend in the pitch direction in response to the landscape where the body is situated (Figure 2B).

Each leg base is connected to the body trunk with a rotational actuator to generate swing motion in a forward-backward direction. In contrast, a linear actuator is implemented at the distal part of the leg to generate lifting and lowering motions (Figure 2A). These rotational and linear actuators produce the torque and force to realize the target angle $\left({\bar{\theta }}_{i}\right)$ and target length $\left({\bar{l}}_{i}\right)$ using proportional-derivative control. In addition, a previous study suggested that centipede legs were likely to behave flexibly against the external forces from the environment that impede propulsion (Ozkan-Aydin et al., 2020). To simplify the passive mechanics of the leg in our model, we implemented a hinge joint between the leg base and tip (Figure 2C). Specifically, we modeled this joint such that the leg bends passively when the leg-tip receives an external force from the front (e.g., the reaction force from front obstacles). In contrast, it stiffens to prevent bending when the leg-tip receives an external force from behind (e.g., ground reaction force while kicking the ground). The details of the mechanical system are described in the Materials and Methods section (Eqs 9–12) and Supplementary Material.

### 3.2 Control System

**FIGURE 3:**
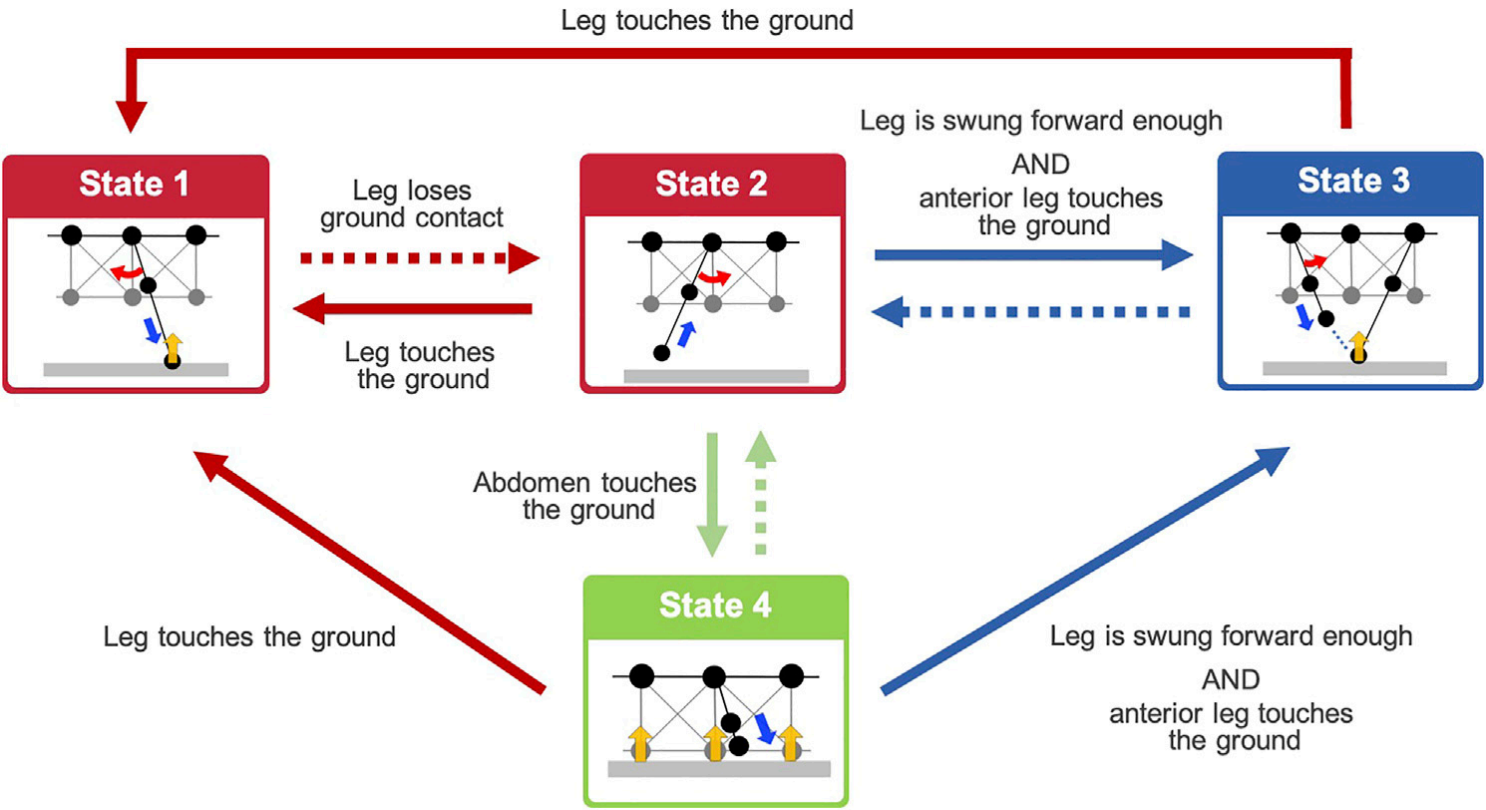
Overview diagram of the behavioral transitions based on the proposed decentralized leg control rules. Dashed arrows denote the negation of conditions represented by the same colored arrows. States 1–4 denote the action executed by Rules 1–4, respectively. Details of the action in each state are illustrated in Figure 4.

From the behavioral findings described in Section 2, we hypothesized that the adaptive walking behavior of the centipedes emerges through the following control rules (see Figure 3):

Rule 1: When a leg makes contact with the ground (red arrows in Figure 3), it swings backward, pushing itself against the ground (State 1).
Rule 2: When a leg loses the ground contact (red dashed arrow in Figure 3), it lifts and swings forward and then stops at the position near the middle of the body segment (State 2).
Rule 3: A leg in the swing phase begins moving to trail the ground contact point of the anterior leg (State 3) if the leg is swung forward enough, and its nearest anterior leg establishes contact with the ground (blue arrows in Figure 3).
Rule 4: When the ventral body surface touches the ground (green arrow in Figure 3), the leg near the contacted body part lowers itself against the ground (State 4).

To describe the abovementioned control rules mathematically, here we introduced a variable *χ*_*i*_, which indicates the activation of Rule 3 and takes the binary values of 0 (inactivated) and 1 (activated). Depending on the value of *χ*_*i*_, our model switches the target angle $\left({\bar{\theta }}_{i}\right)$ and target length $\left({\bar{l}}_{i}\right)$ of the *i*-th leg as follows:

$$\begin{array}{ccc} {\bar{\theta }}_{i} & = & \left(1-\chi_{i}\right){\bar{\theta }}_{\alpha ,i}+\chi_{i}{\bar{\theta }}_{\beta ,i}, \end{array}$$ (1)

$$\begin{array}{ccc} {\bar{l}}_{i} & = & \left(1-\chi_{i}\right){\bar{l}}_{\alpha ,i}+\chi_{i}{\bar{l}}_{\beta ,i}. \end{array}$$ (2)

**FIGURE 4:**
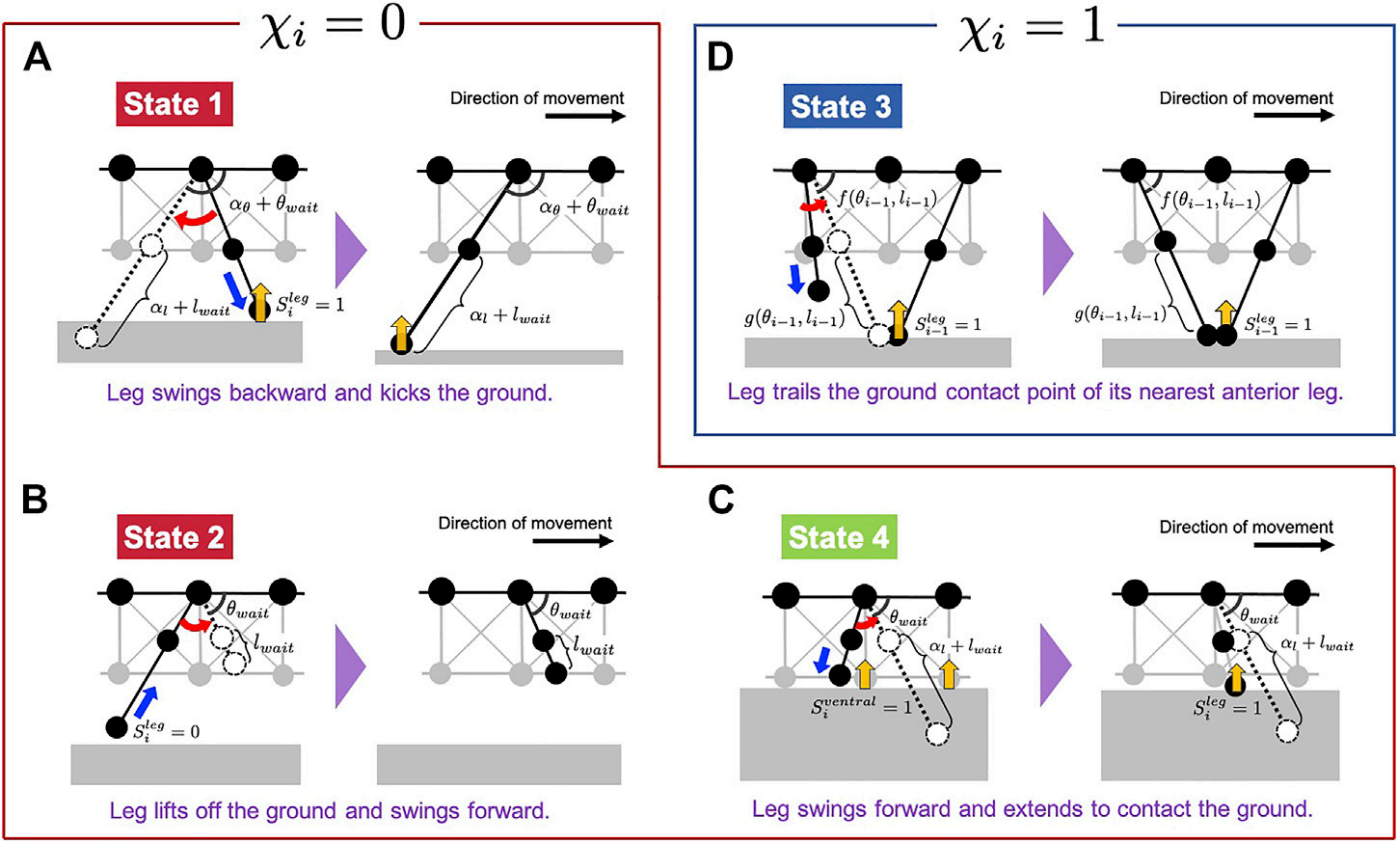
Schematic of the leg control rules proposed in our model. Subfigures **(A–D)** correspond to States 1, 2, 4, and 3, respectively. The legs, illustrated in dashed lines, denote the target positions in each situation.

When *χ*_*i*_ = 0, Rule 3 is not activated (i.e., the state of the leg controller corresponds to either Rule 1, 2 or 4), and ${\bar{\theta }}_{i}={\bar{\theta }}_{\alpha ,i}$ and ${\bar{l}}_{i}={\bar{l}}_{\alpha ,i}$ . In contrast, when *χ*_*i*_ = 1, Rule 3 is activated and ${\bar{\theta }}_{i}={\bar{\theta }}_{\beta ,i}$ and ${\bar{l}}_{i}={\bar{l}}_{\beta ,i}$ . Each target position $\left({\bar{\theta }}_{\alpha ,i},{\bar{l}}_{\alpha ,i},{\bar{\theta }}_{\beta ,i},{\bar{l}}_{\beta ,i}\right)$ is formulated below (Figure 4).

Specifically, *χ*_*i*_ is defined as follows:

$$\chi_{i}=\left\{\begin{array}{cc} S_{i-1}^{leg}\left(1-S_{i}^{leg}\right)U\left(\theta_{FCP}-\theta_{i}\right)\; & \left(i\neq 1\right)\\ \left(1-S_{i}^{leg}\right)U\left(\theta_{FCP}-\theta_{i}\right)\; & \left(i=1\right), \end{array}\right.$$ (3)

where in $S_{i}^{leg}$ denotes the ground contact signal detected at the *i*-th leg-tip and takes the binary value of 0 and 1. The function *U*(⋅) is a unit step function and *θ*_*FCP*_ is the positive constant which indicates the threshold value of the leg angle. Thus, Eq. 3 means that if the *i*-th leg is in the swing phase $\left(S_{i}^{leg}=0\right)$ swung forward enough (*U*(*θ*_*FCP*_ − *θ*_*i*_) = 1), and the nearest anterior leg comes into contact with the ground $\left(S_{i-1}^{leg}=1\right)$ , then *χ*_*i*_ becomes 1 and Rule 3 is activated. As for the first leg (*i* = 1), we assumed that *χ*_1_ becomes 1 without the condition of ground contact of its anterior leg.

First, we explain the formulation of Rules 1, 2 and 4 when *χ*_*i*_ = 0 (Figures 4A–C). The target angle $\left({\bar{\theta }}_{\alpha ,i}\right)$ and target length $\left({\bar{l}}_{\alpha ,i}\right)$ of the *i*-th leg are described as follows:

$$\begin{array}{ccc} \tau_{\theta }{\dot{\bar{\theta }}}_{\alpha ,i}=-{\bar{\theta }}_{\alpha ,i}+\theta_{wait}+\alpha_{\theta }S_{i}^{leg}, & & \end{array}$$ (4)

$$\begin{array}{ccc} {\bar{l}}_{\alpha ,i}=l_{wait}+\alpha_{l}\left\{\max\left(S_{i}^{leg},S_{i}^{ventral}\right)\right\}, & & \end{array}$$ (5)

where *τ*_*θ*_ is the time constant, *θ*_*wait*_ and *l*_*wait*_ denote the target leg position during swing phase, and *α*_*θ*_ and *α*_*l*_ denote the amplitude of the leg motion during stance phase. $S_{i}^{ventral}$ denotes the ground contact signal detected at the ventral surface of the *i*-th body segment and takes the binary values of 0 and 1. More precisely, when either the mass point just below the leg base (“*VC*_*i*_” in Figure 2A) or the mass point before it (“*VB*_*i*_” in Figure 2A), touches the ground, $S_{i}^{ventral}$ becomes 1. When the *i*-th leg has ground contact $\left(S_{i}^{leg}=1\right)$ , it is controlled to swing backward $\left({\bar{\theta }}_{\alpha ,i}\to \alpha_{\theta }+\theta_{wait}\right)$ and push the tip against the ground $\left({\bar{l}}_{\alpha ,i}=\alpha_{l}+l_{wait}\right)$ . Thus, this is the case of Rule 1 (Figure 4A). Next, when neither the leg nor the body trunk have ground contact $\left(S_{i}^{leg}=0\wedge S_{i}^{ventral}=0\right)$ , the leg lifts up $\left({\bar{l}}_{\alpha ,i}=l_{wait}\right)$ and swings forward until it reaches the resting position $\left({\bar{\theta }}_{\alpha ,i}\to \theta_{wait}\right)$ . This case corresponds to Rule 2 (Figure 4B). Finally, when only the ventral body surface touches the ground $\left(S_{i}^{leg}=0\wedge S_{i}^{ventral}=1\right)$ , the leg swings forward $\left({\bar{\theta }}_{\alpha ,i}\to \theta_{wait}\right)$ and lowers the tip to touch the ground $\left({\bar{l}}_{\alpha ,i}=\alpha_{l}+l_{wait}\right)$ . Thus, Rule 4 (Figure 4C) is also implemented in the Eqs 4, 5.

Next, we explain the formulation of Rule 3 when *χ*_*i*_ = 1 (Figure 4D). The target angle $\left({\bar{\theta }}_{\beta ,i}\right)$ and target length $\left({\bar{l}}_{\beta ,i}\right)$ of the *i*-th leg are described below.

$${\bar{\theta }}_{\beta ,i}=\left\{\begin{array}{cc} f\left(\theta_{i-1},l_{i-1}\right)\; & \left(i\neq 1\right)\\ \theta_{front}\; & \left(i=1\right), \end{array}\right.{\bar{l}}_{\beta ,i}=\left\{\begin{array}{cc} g\left(\theta_{i-1},l_{i-1}\right)\; & \left(i\neq 1\right)\\ l_{front}\; & \left(i=1\right). \end{array}\right.$$ (6)

herein, *f*(*θ*_*i*−1_, *l*_*i*−1_) and *g*(*θ*_*i*−1_, *l*_*i*−1_) are functions which determine the target position of the *i*-th leg using inverse kinematics, to enable the leg-tip position to match that of its nearest anterior leg (Figure 4D). Details of these functions are shown in Materials and Methods section (Eqs 13, 14). *θ*_*front*_ and *l*_*front*_ are positive constants, and we assumed that the first leg (*i* = 1) swings forward and lowers its tip to realize the constant target position in Rule 3.

## 4 Simulation Results

To evaluate our proposed model, we conducted walking experiments in three different environments using simulation: (1) on flat terrain, (2) on terrain with evenly spaced gaps, and (3) on irregular terrain with randomly placed obstacles. Tables 1, 2 list the respective values of body parameters and control parameters employed in the simulations. These parameters were chosen by trial and error according to the following criteria: the mechanical parameters corresponding to the softness of the trunk and leg were tuned such that the trunk and legs can passively bend moderately when they receive reaction force from the environment. As for the control parameters for the leg motions, we tuned them such that the wavelength of the retrograde wave gait and the locomotion speed on flat terrain roughly match those of real centipedes. The program for simulation was written in C++ and the simulation results were visualized using OpenGL. The differential equations were solved using the fourth-order Runge-Kutta method with a time step of 4.0 × 10^−6^ s.

**TABLE 1:** Body parameter values employed in the simulations.

Parameter	Value	Value for model A	Dimension
Number of legs	21		
Total body length	8.6 × 10^−2^		[m]
Total body mass	2.88 × 10^−3^		[kg]
*k* ^*body*^	8.0 × 10^−4^	→ 8.0 × 10^−2^	[m^2^s^−2^kg]
*d* ^*body*^	8.0 × 10^−7^		[m^2^s^−1^kg]
$k_{rigid}^{hinge}$	8.0 × 10^−4^		[m^2^s^−2^kg]
$k_{soft}^{hinge}$	8.0 × 10^−6^	→ 8.0 × 10^−4^	[m^2^s^−2^kg]
*d* ^*hinge*^	8.0 × 10^−7^		[m^2^s^−1^kg]

**TABLE 2:** Control parameter values employed in the simulations.

Parameter	Value	Dimension
*τ*_*θ*_	1.5 × 10^−1^	[s]
*α*_*θ*_	1.6	
*θ*_*wait*_	2*π*/7	
*α*_*l*_	4.4 × 10^−3^	[m]
*l*_*wait*_	1.0 × 10^−3^	[m]
*θ*_*FCP*_	*π*/2	
*θ*_*front*_	*π*/4	
*l*_*front*_	3.04 × 10^−3^	[m]
*k* ^*r*^	4.8 × 10^−5^	[m^2^s^−2^kg]
*d* ^*r*^	8.0 × 10^−7^	[m^2^s^−1^kg]
*k* ^*l*^	1.2 × 10^1^	[s^−2^kg]
*d* ^*l*^	5.0 × 10^−1^	[s^−1^kg]
Parameter for Model B		
*ω*	16.5	[s^−1^]
*ψ*	2*π*/5	
*α* ^*B*^	3*π*/14	
*β* ^*B*^	4.4 × 10^−3^	[m
*L* ^*B*^	5.4 × 10^−3^	[m]

### 4.1 Stable Walking on Flat Terrain

**FIGURE 5:**
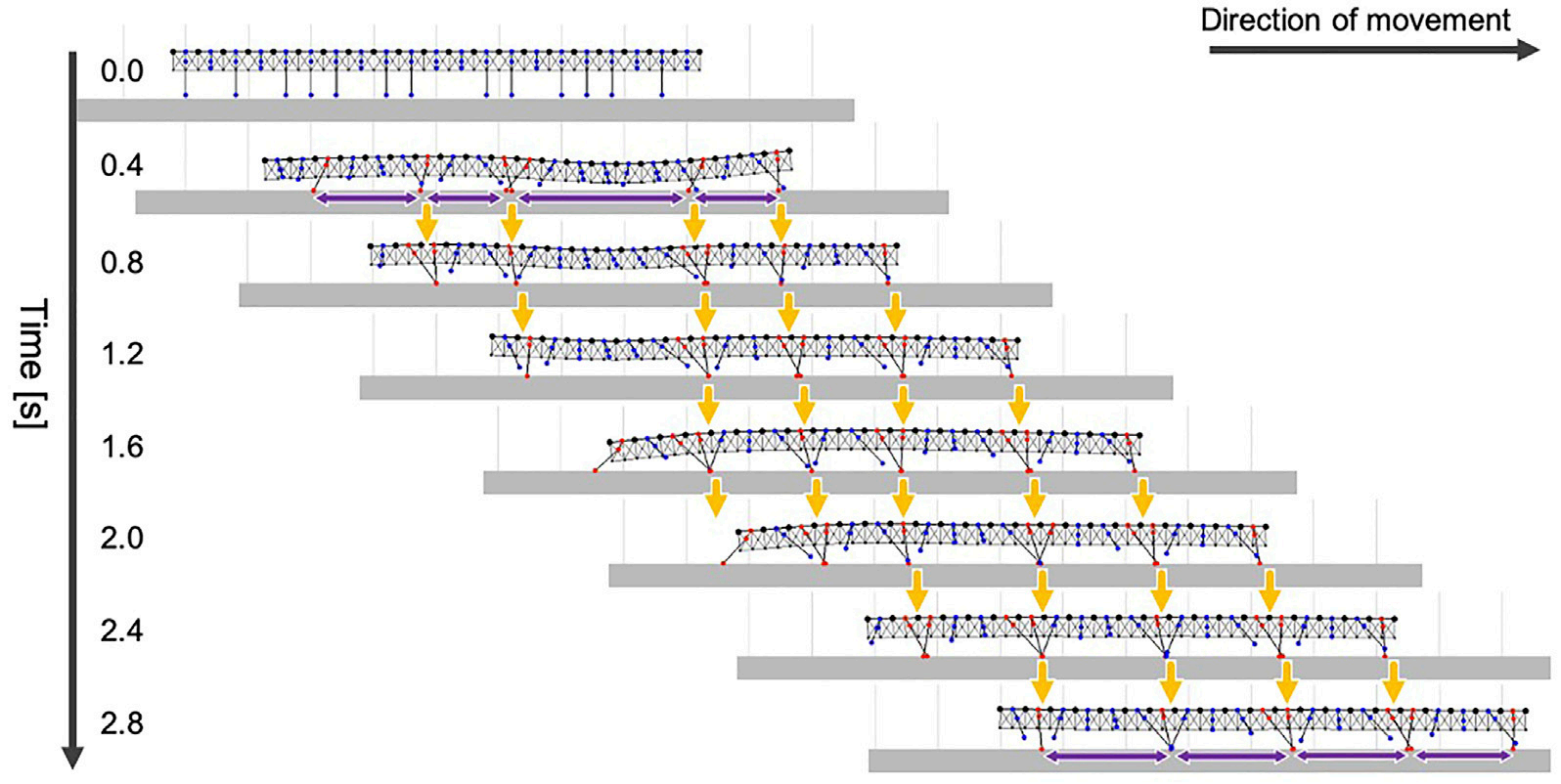
Snapshots of the simulated centipede walking on flat terrain using the proposed model. Orange arrows indicate that the ground contact points of the anterior legs were trailed by the posterior legs. Purple arrows denote the wavelength of the leg movements.

First, we tested whether our model could produce the typical gait pattern of the centipede on flat terrain. The initial leg positions of the simulated centipede were set to be varied as follows: eight legs (*i* = 1, 3, 7, 10, 11, 14, 18, 20) were lifted up (*l*_*i*_ = *l*_*wait*_, $\theta_{i}=\frac{\pi }{2}$ ) and the other thirteen legs were extended (*l*_*i*_ = *l*_*wait*_ + *α*_*l*_, $\theta_{i}=\frac{\pi }{2}$ ). Snapshots and the movie of simulated walking are shown in Figure 5 and Supplementary Movie S3. The simulated centipede immediately exhibited walking in which each leg trailed the ground contact point of its nearest anterior legs, and the wave of leg movement propagated from the head to the tail. Furthermore, although we varied the initial leg positions, the gait pattern spontaneously converged to the wavelength (approximately five legs) similar to the behavioral finding in centipedes (Manton, 1965). Thus, we confirmed that our model can self-organize the typical gait of centipedes on flat terrain through the decentralized control rules.

### 4.2 Adaptive Walking on Uneven Terrain With Gaps

**FIGURE 6:**
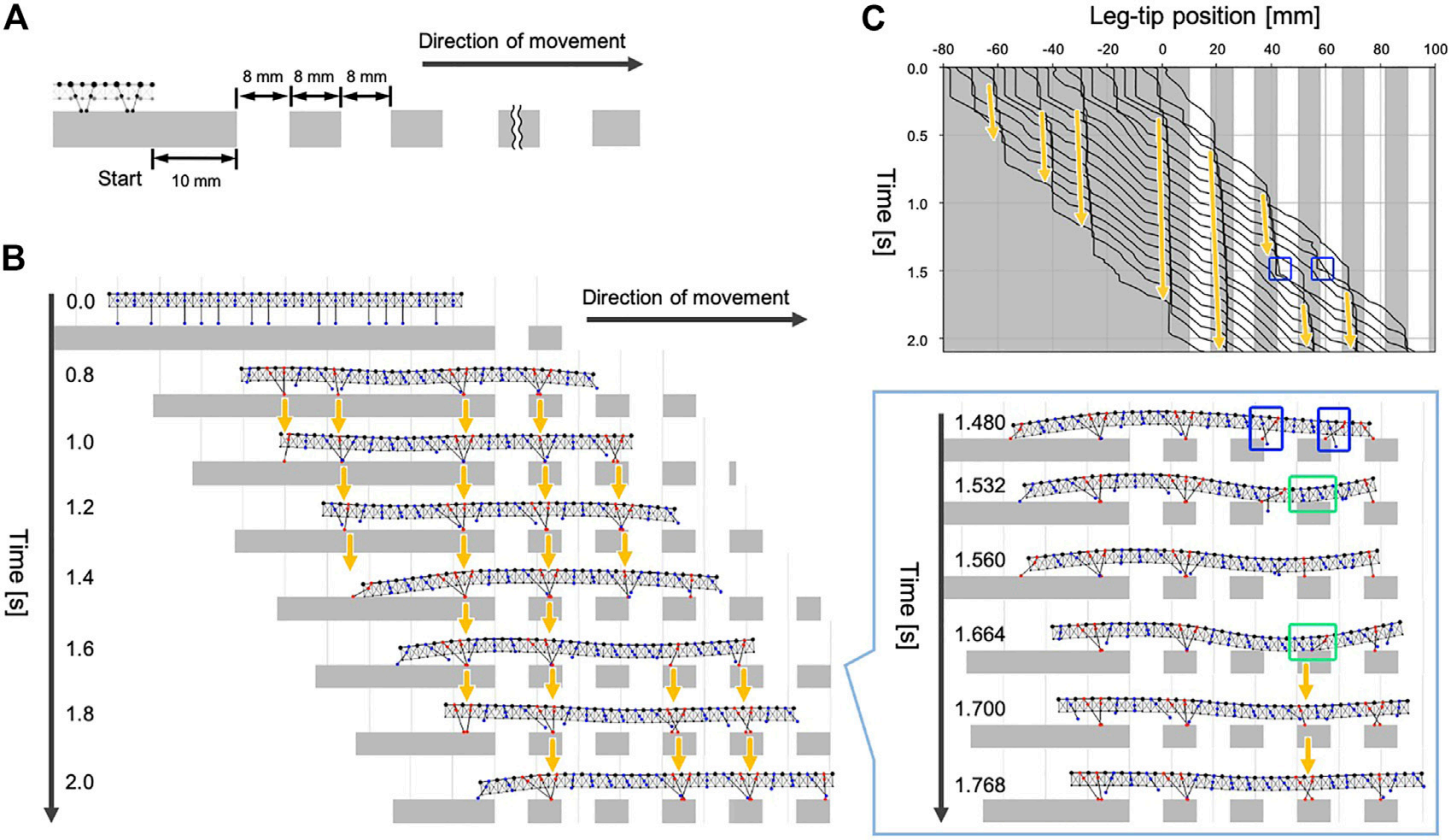
Simulation results for walking on terrain with evenly spaced gaps. **(A)** Schematic of the experimental setup. **(B)** Snapshots of the simulated walking using the proposed model. **(C)** Spatiotemporal plots of the leg-tip positions. Orange arrows denote the ground contact points of the anterior legs were trailed by their neighboring posterior legs. Blue squares indicate the legs missed the footing. Green square indicates the body section where the ventral surface touched the ground.

Next, we tested whether our model could generate adaptive gaits when the available scaffolds are limited. Specifically, we prepared walking terrain with evenly spaced gaps, as shown in Figure 6A. The width of each gap was approximately one-tenth of the total body length. The initial leg positions were the same as the experiment in Section 4.1, and the simulated centipede started walking from the flat terrain in front of the gap areas.

The snapshots and the movie of the simulated walking are shown in Figure 6B and Supplementary Movie S4. When the simulated centipede entered the terrain with gaps, the first leg obtained a foothold due to the passive body bending. Subsequently, the following posterior legs moved to roughly trail the ground contact point of the first leg (see the period 0.8–1.4 s, Figure 6B). Thus, our model could produce the characteristic centipede gait even on the terrain with many gaps. Furthermore, we found that the simulated centipede could instantly recover from the missed footing during walking (see the period 1.4–1.8 s, Figure 6B). In our model, the legs sometimes failed to obtain footholds around the edge of the ground because Rule 3 did not accurately control the leg position to trail the ground contact point of its anterior leg. However, once the legs missed footholds (1.48 s), the body trunk near the legs passively bent and touched the ground (1.532 and 1.664 s). This ground contact signal induced the neighboring legs to lower their tips to obtain the foothold by Rule 4 (1.56 and 1.7 s). Consequently, the posterior legs in the swing phase successfully trailed the newly obtained foothold in front (1.7–2.0 s). Such behavioral sequences qualitatively match the third behavioral finding in Section 2. Thus, the results suggest that our model can generate adaptive gaits on uneven terrain through the interactions between decentralized leg control, flexible body dynamics, and the environment.

It should be noted that our behavioral experiment in Section 2 and the simulated experiment in this section were not under the same conditions. In the behavioral experiment, when we restored the scaffold at the gap area, it was brought back to the height where the leg tips actually make contact. In contrast, the positions of the scaffolds in this simulation experiment were fixed, so after losing foothold, the legs did not always make contact with the scaffold, depending on the extent of passive bending of the trunk. In the case of Figure 6B, the legs at two areas missed its footing simultaneously (indicated in blue squares) and the front part obtained the scaffold first. Consequently, at the hind part, the trunk bending was suppressed and the legs did not obtain scaffold again.

### 4.3 Evaluating Walking Performance on Irregular Terrains With Obstacles

In the final simulation, we quantitatively evaluated the walking performance of our model on irregular terrain. Note that the main focus here was to verify adaptive leg control improved walking performance when it was well-coupled with the passive dynamics of flexible bodies. Therefore, we compared the walking performance of our proposed model with that of two different configurations (Table 3): (1) when the body was rigid (Model A), and (2) when the adaptive leg control was eliminated (Model B).

**TABLE 3:** Characteristics of the models used for comparison.

	Body Flexibility	Adaptive leg Control
Proposed model	+	+
Model A	−	+
Model B	+	−

#### 4.3.1 Different Model Configurations for Comparison

In this experiment, we used the same body and control parameters as in Sections 4.1, 4.2 for the proposed model. The different model configurations we prepared to compare the walking performance are as follows:

Model A: To eliminate the effect of body flexibility from our model, we stiffened the passive torsional springs at the dorsal side of the body by changing their spring constant to a larger value (*k*_*body*_). In addition, we replaced the hinge joints at the legs (Figure 2C) with rigid joints that always prevented the leg from bending against the external forces. Specifically, we stiffened the flexible torsional springs implemented around the joint (see Eq. 12; Table 1). The remainder of the model settings and parameters were the same.
Model B: To eliminate the effect of adaptive leg control from our model, we implemented an open-loop controller that produces a fixed gait. Specifically, we used the retrograde wave gait and set the target angle $\left({\bar{\theta }}_{i}\right)$ and target length $\left({\bar{l}}_{i}\right)$ of the *i*-th leg as follows:

$$\begin{array}{ccc} {\bar{\theta }}_{i} & = & \frac{\pi }{2}+\alpha^{B}\cos\left(\omega t-\psi i\right), \end{array}$$ (7)

$$\begin{array}{ccc} {\bar{l}}_{i} & = & L^{B}-\beta^{B}\max\left[\sin\left(\omega t-\psi i\right),0\right], \end{array}$$ (8)

where *ω* and *ψ* denote the respective walking frequency and phase difference between the adjacent legs, and *α* ^*B*^, *L* ^*B*^ and *β* ^*B*^ are the positive constants. These parameter values are listed in Table 2. In principle, it is difficult to design an open-loop controlled gait equal to that of our proposed model. Thus, we chose these control parameters so that the walking speed, the wavelength, and the leg trajectory approximately matched the result on flat terrain in Section 4.1.

#### 4.3.2 Experimental Setup and Evaluation Methods

**FIGURE 7:**
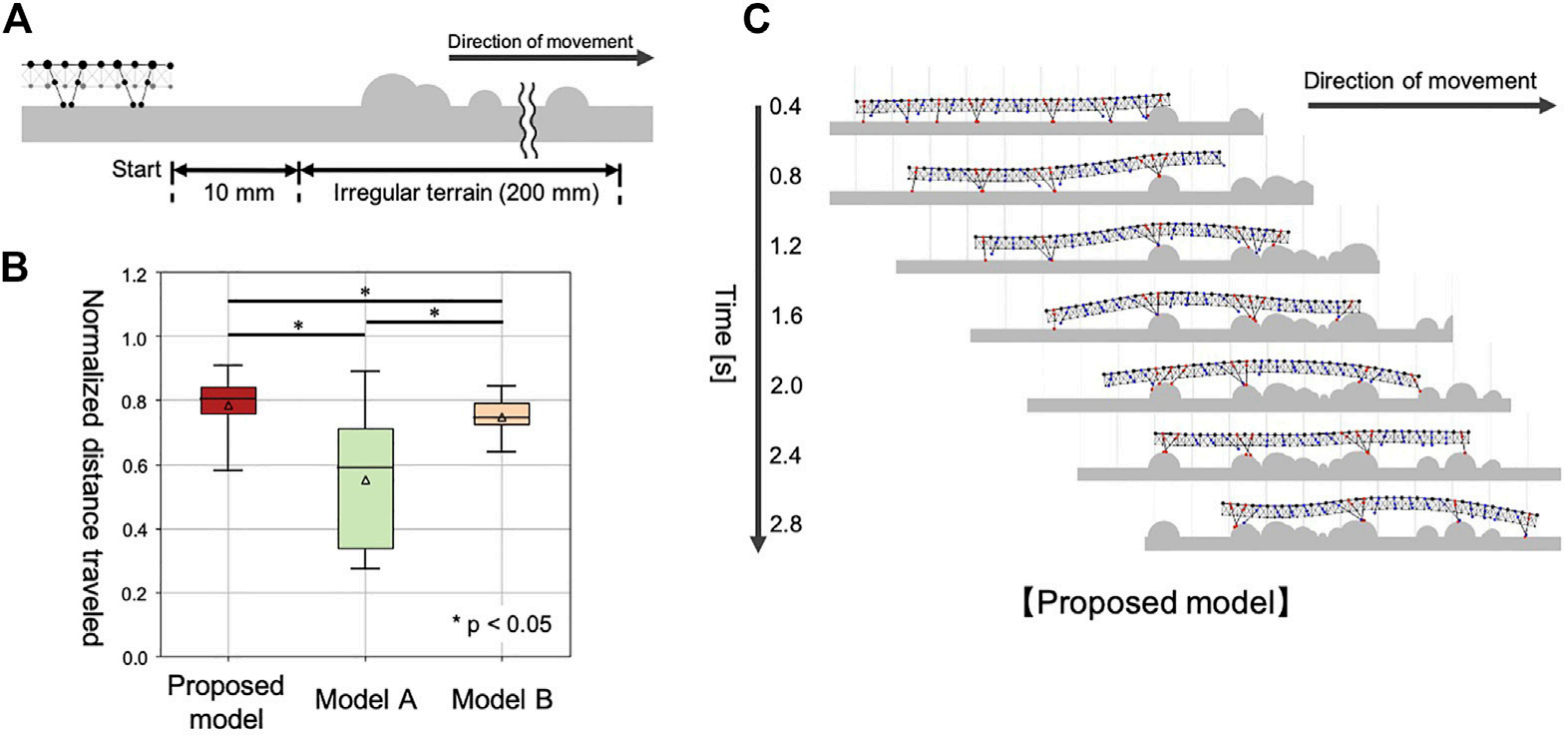
Simulation results for walking on irregular terrain with many obstacles. **(A)** Schematic of the experimental setup. **(B)** Comparison of the walking performance with different models. **(C)** Snapshots of the representative simulated walking using our proposed model.

We prepared a test environment consisting of flat terrain followed by 20 cm of irregular terrain (Figure 7A). Irregular terrains were modeled by setting 30 semicircular obstacles, with radii ranging from 0 to 5 mm, randomly placed on the ground. For statistical analysis, we used 30 variations of the irregular terrain. In each test environment, the simulated centipede started walking from a position 10 mm in front of the irregular terrain, and the distance traveled in 3 seconds was measured. To compare the different models fairly, each traveled distance in the test environment was normalized by the 30 trial average of the traveled distances on flat terrain in 3 sec, using the corresponding model (Figure 7B). Each model was simulated once for each test environment (irregular terrain). The initial positions of the legs were set randomly for every trial in this experiment.

#### 4.3.3 Results

**FIGURE 8:**
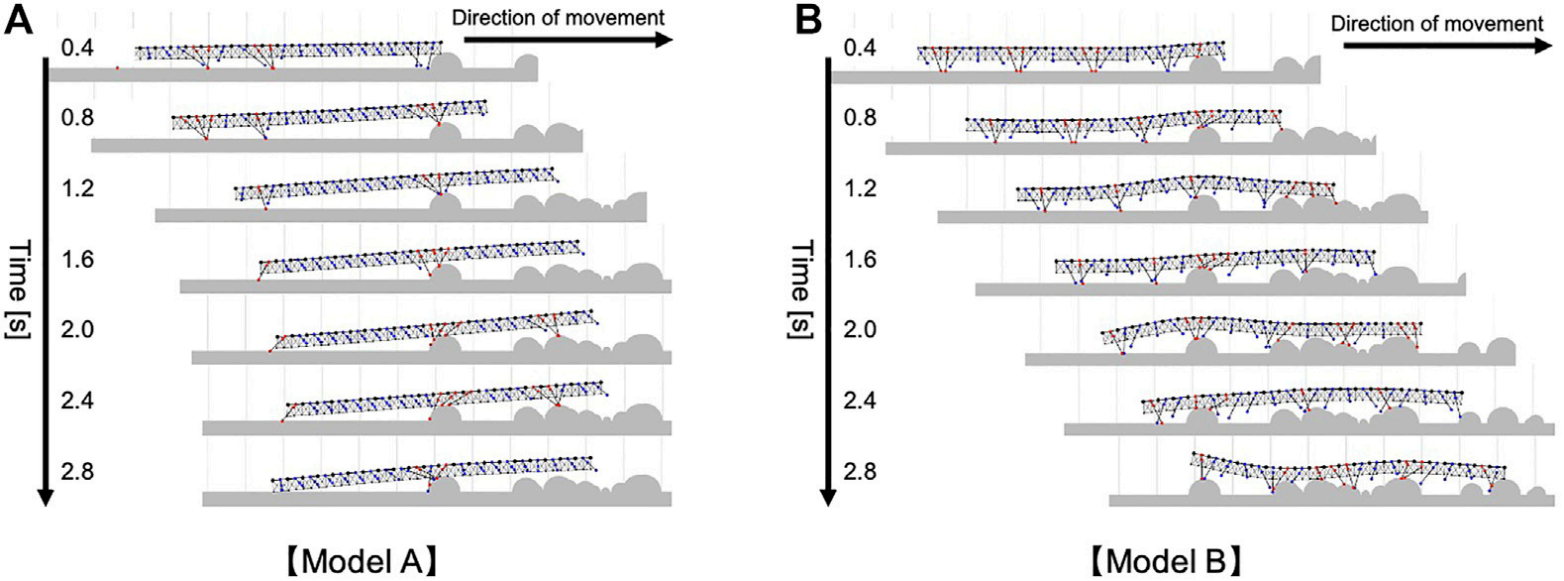
Snapshots of the representative simulated walking on irregular terrain with many obstacles **(A)** when Model A was implemented, **(B)** when Model B was implemented. These trials were conducted in the same walking environment with Figure 7C.

The compared walking performance on irregular terrains is summarized in Figure 7B (Supplementary Movie S5). Multiple comparison tests were conducted using the Holm method. The results indicated our proposed model significantly outperformed the two different models (Model A and B) in terms of the distance traveled (*p* < 0.05). As shown in Figure 7C, the simulated centipede using our proposed model exhibited adaptive and effective walking, in which the legs trailed the scaffolds obtained at the anterior body section. In contrast, when using Model A, the simulated centipede had difficulty obtaining scaffolds due to the rigid bodies (Figure 8A). Model B achieved relatively successful walking because it exploited the flexible body dynamics that enabled the adaptation to the terrain irregularities (Figure 8B). However, depending on the landscape, the legs sometimes swung backward in the air due to the fixed gait. Thus, these results suggest that our proposed model achieved more effective walking on irregular terrain through the synergetic coupling of the flexible body dynamics and the adaptive leg control.

## 5 Discussion

The significance of this study is that we have proposed a model that combines adaptive leg control with flexible body dynamics in centipede walking. From the behavioral experiment using *Scolopendra subspinipes mutilans*, we found that each leg can spontaneously adapt to environmental changes (e.g., suddenly appearing and disappearing scaffolds) and uses the walking strategy of trailing the ground contact point of anterior legs. Drawing inspiration from this adaptive leg motion, we have proposed decentralized control mechanisms that exploit the ground contact sense of the leg-tip and the ventral body surface. More specifically, compared to our previous models (Yasui et al., 2017a; Yasui et al., 2017b), Rules 3 and 4 are the newly implemented control algorithms in this study. Using our model, we demonstrated that on irregular terrain, the simulated centipede exhibited the highest performance when the adaptive leg control and flexible body dynamics both worked well. Thus, our model contributes to enhancing the locomotor performance of multi-legged robots in unstructured environments, as well as understanding the essential control mechanisms underlying adaptive centipede walking.

### 5.1 Biological Implications for Control Mechanisms in Multi-Legged Walking

Our modeling study has implications for the biological understanding of control mechanisms underlying myriapod walking. Our simulation results suggest that decentralized leg control for trailing the ground contact point of the nearest anterior leg (i.e., Rule 3) realizes effective walking by exploiting the limited secure scaffolds obtained in front (Sections 4.2, 4.3). Although the actual physiological mechanisms for this control are yet to be clarified, related mechanisms have been reported in insects (Wendler, 1966; Wong and Pearson, 1976; Dean and Schmitz, 1992; Theunissen et al., 2014). For example, in cockroaches, the ablation of a hair plate at the most proximal leg joint causes the operated leg to overstep and collide with its ipsilateral anterior leg (Wong and Pearson, 1976). Furthermore, in stick insects, such ablation of the hair plate affects not only the motion of the operated leg, but also the spatial coordination of the adjacent legs (Dean and Schmitz, 1992). These findings indicate that the proprioceptive signals are shared among neighboring legs and are essential for establishing the appropriate spatial interlimb coordination. Thus, similar mechanisms may also exist in centipedes for the abovementioned kinematic leg control.

In addition, our model predicted on the control mechanism centipedes may exploit using the ground contact sense detected at the ventral side of the body trunk, for adaptive walking. Specifically, we have proposed a hypothesized control rule, which states that when the ventral body surface contacts the ground, the legs near the contacted point start to lower their leg-tips to obtain footholds (i.e., Rule 4). This mechanism seems reasonable because it allows the animal to instantly recover from the failure of the foot placement (Section 4.2) or adapt its walking motion to unpredictable sudden changes in the walking terrain. As suggested from the simulation results using rigid bodies (Model A in Section 4.3 and Figure 8A), this mechanism works more effectively when it is coupled with the flexible body. This is because the passive dynamics of the flexible body enable the postural changes along the landscape of irregular terrain, which leads to easier obtainment of ground contact at the ventral body surface. Although this control mechanism (Rule 4) has not been examined through biological experiments, it was reported in stick insects that they exhibited rhythmic leg movement in the vertical plane in the absence of ground contact of a leg when tactile stimulation was given at the abdominal surface (Berg et al., 2015). Thus, we expect that our proposed leg control mechanism using abdominal contact may exist in centipedes.

Similar rule-based modeling approaches for the arthropod walking can also be seen in the insect studies. For example, Cruse et al. (1998) proposed decentralized control rules for hexapod walking from the experimental results of stick insects. Their representative model “Walk-net” (Schilling et al., 2013), which consisted of simple reflexive rules for each leg motions, could successfully reproduce the adaptive insect gaits (Wilson, 1966). It should be emphasized that their model did not assume the intrinsic rhythm generators for walking motions, such as central pattern generators (Ijspeert, 2008). As for this point, our proposed centipede model and the Walk-net share essentially similar control frameworks. Indeed, we believe that sensory inputs related to the ground contact play a crucial role for shaping walking rhythms, because, during gap crossing, centipedes’ legs over the gap stopped periodic motions (Yasui et al., 2017b). Further comparative studies of hexapods and myriapods will help explain the common control principles underlying multi-legged walking of arthropods.

### 5.2 Limitations of This Study and Future Works

This study still has some limitations. First, the simulated centipede using the proposed model sometimes missed footing on the ground depending on its body posture and the landscape, which led to the walking performance with relatively large variance (as shown in Figure 7B). One possible reason for this problem is that the leg control rule for trailing the contact point of the anterior leg (i.e., Rule 3), was not a precisely calculated control (see the Materials and Methods section). Thus, it may be useful from the engineering perspective to modify our model with more precise position control for Rule 3. Also, it would be of interest to investigate how accurately centipedes control each leg to trail the anterior ground contact points to achieve their high locomotor performance.

Secondly, our 2D model in a sagittal plane cannot examine the 3D aspects of the centipede locomotion. Although we consider our 2D model captures the essence of the dynamic interaction between the flexible body and environment, centipedes may change the left-right coordination between the legs in response to the landscape and 3D body dynamics should affect the locomotion when they move on more complex terrain. Therefore, investigating the locomotor transitions of centipedes on 3D unstructured terrain and extending our model to 3D will provide deeper insights into biology and robotics.

Thirdly, evaluating the locomotor performance of our model in more diverse and complex terrain is required in the future. For example, it is important to validate the adaptability to changes in physical properties of the ground such as slipperiness and stiffness. However, it is difficult to simulate these conditions accurately because many of the physical parameters are unknown. To overcome this limitation, testing models in real-world environments using robotic platforms (Aguilar et al., 2016; Gravish and Lauder, 2018), would be an effective approach.

Finally, we briefly discuss potential future research directions toward fully understanding the high locomotor performance of centipedes in complex environments. As our main focus was on the lower locomotor circuits that control each leg’s motions during walking, we have not considered the role of descending control from the higher centers (the brain) and the active body movement near the head section. However, it must be essential for navigating complex terrain that the head detects the surrounding environmental situation using antennal sensing and appropriately controls the movement of the anterior body section. Therefore, integrating our model with an active head control is an important next step.

## 6 Materials and Methods

### 6.1 Behavioral Experiments

We used four centipedes (*Scolopendra subspinipes mutilans*), which were wild-caught in Wakayama, Japan. The body length of the subjects was 9.1 ± 0.4 cm. Observations were recorded from the top and side views using two high-speed video cameras (DITECT, type HAS-U2) at a resolution of 800 × 600 pixels and a frame rate of 300 frames per second (Figure 1A). Spatiotemporal plots of the leg positions (Figure 1C) were produced using the image processing software, ImageJ. The experiment was conducted for 25 trials in total and qualitatively similar behaviors were observed.

### 6.2 Model

To realize the target angle $\left({\bar{\theta }}_{i}\right)$ and target length $\left({\bar{l}}_{i}\right)$ of each leg, the torque $\left(\tau_{i}^{leg}\right)$ and force $\left(f_{i}^{leg}\right)$ , actively generated by the torsional and linear actuators, are determined according to the proportional-derivative control as follows.

$$\begin{array}{ccc} \tau_{i}^{leg}=-k^{r}\left(\theta_{i}-{\bar{\theta }}_{i}\right)-d^{r}{\dot{\theta }}_{i}, & & \end{array}$$ (9)

$$\begin{array}{ccc} f_{i}^{leg}=-k^{l}\left(l_{i}-{\bar{l}}_{i}\right)-d^{l}\dot{l_{i}}, & & \end{array}$$ (10)

where *θ*_*i*_ and *l*_*i*_ are the actual angle and length of each leg actuators, respectively, and *k* ^*r*^, *k* ^*l*^, *d* ^*r*^, *d* ^*l*^ are the positive constants.

The passive torque $\left(\tau_{i}^{body}\right)$ generated due to the dorsal flexibility of each body trunk (shown in Figure 2B) is described as follows:

$$\tau_{i}^{body}=-k^{body}\left(\theta_{i}^{body}-\pi \right)-d^{body}{\dot{\theta }}_{i}^{body},$$ (11)

where $\theta_{i}^{body}$ is the actual bending angle of the body trunk and *k* ^*body*^ and *d* ^*body*^ are the spring constant of the passive rotational spring and the damping coefficient, respectively. Meanwhile, the passive torque $\left(\tau_{i}^{hinge}\right)$ generated due to the anisotropic flexibility of the leg hinge joint (shown in Figure 2C) is described as follows:

$$\tau_{i}^{hinge}=\left\{\begin{array}{cc} -k_{rigid}^{hinge}\left(\theta_{i}^{hinge}-\pi \right)-d^{hinge}{\dot{\theta }}_{i}^{hinge}\; & \left(when\;\theta_{i}^{hinge}<\pi \right)\\ -k_{soft}^{hinge}\left(\theta_{i}^{hinge}-\pi \right)-d^{hinge}{\dot{\theta }}_{i}^{hinge}\; & \left(when\;\theta_{i}^{hinge}\geq \pi \right), \end{array}\right.$$ (12)

where $k_{rigid}^{hinge}$ and $k_{soft}^{hinge}$ are the spring constants of the passive rotational spring and *d* ^*hinge*^ is the damping coefficient. Thus, we implemented the anisotropic flexibility by changing the spring constants $\left(k_{rigid}^{hinge}\gg k_{soft}^{hinge}\right)$ depending on the actual angle of the hinge joint $\left(\theta_{i}^{hinge}\right)$ .

The target angle $\left({\bar{\theta }}_{\beta ,i}\right)$ and target length $\left({\bar{l}}_{\beta ,i}\right)$ of the *i*-th leg (*i* ≠ 1) in Rule 3 are determined using the following functions *f*(*θ*_*i*−1_, *l*_*i*−1_) and *g*(*θ*_*i*−1_, *l*_*i*−1_), respectively:

$$\begin{array}{ccc} f\left(\theta_{i-1},l_{i-1}\right) & = & \arcsin\frac{\left(l_{i-1}+l_{cf}\right)\sin\theta_{i-1}}{g\left(\theta_{i-1},l_{i-1}\right)+l_{cf}}, \end{array}$$ (13)

$$\begin{array}{ccc} g\left(\theta_{i-1},l_{i-1}\right) & = & \sqrt{{l_{body}}^{2}+{\left(l_{i-1}+l_{cf}\right)}^{2}+2l_{body}\left(l_{i-1}+l_{cf}\right)\cos\theta_{i-1}}-l_{cf}, \end{array}$$ (14)

where *l*_*cf*_ is the natural length of the passive spring consisting of the proximal part of each leg, and *l*_*body*_ is the positive constant which denotes the distance between the leg bases of the adjacent legs (see Supplementary Figure S2). It should be noted that the calculated target position can deviate from the actual ground contact point of the anterior leg, because our model did not precisely consider the effect of postural changes due to the flexible body trunk.

## Data Availability

The original contributions presented in the study are included in the article/Supplementary Material, further inquiries can be directed to the corresponding author.

## Supplementary Material

Supplementary Movie 1 Adaptive centipede walking from the side view.

Supplementary Movie 2 Adaptive centipede walking from the top view.

Supplementary Movie 3 Simulated walking on flat terrain.

Supplementary Movie 4 Simulated walking on terrain with evenly spaced gaps.

Supplementary Movie 5 Simulated walking on irregular terrain with obstacles.
