# Supplementary material for: Adaptive Centipede Walking via Synergetic Coupling Between Decentralized Control and Flexible Body Dynamics
Source: Front Robot AI. 2022 Apr 5;9:797566. doi: 10.3389/frobt.2022.797566 (PMC9016197; doi:10.3389/frobt.2022.797566)
Supplement: Supplementary file 2 [file DataSheet1.PDF]

Supplementary Material

1 DETAILS OF THE MECHANICAL SYSTEM

In our centipede model, mass points corresponding to the body trunk and proximal part of a leg are connected to each other via five types of links as shown in Figure S1. Each link is modeled by the parallel combination of a passive spring and a damper. The passive forces generated at the ”Dorsal body link” and the “Proximal leg link” (f_i^A), and those at the “Ventral body link”, the “Dorsoventral link”, and the “Diagonal body link” (f_i^B) are described as follows:

$$f_i^A = -k^A(l_i^A - \bar{l}^A)^3 - d^A \dot{l}_i^A, \quad A \in \{Dorsal, Proximal\}, \tag{S1}$$

$$f_i^B = -k^B(l_i^B - \bar{l}^B) - d^B \dot{l}_i^B, \quad B \in \{Ventral, Dorsovent, Diagonal\}, \tag{S2}$$

wherein k^A and k^B are the passive spring constants, and d^A and d^B are the damping coefficients. l_i^A, l_i^B are the actual lengths of the passive spring, and $\bar{l}^A$, $\bar{l}^B$ are the natural lengths of the passive spring. These parameter values are listed in Table S1.

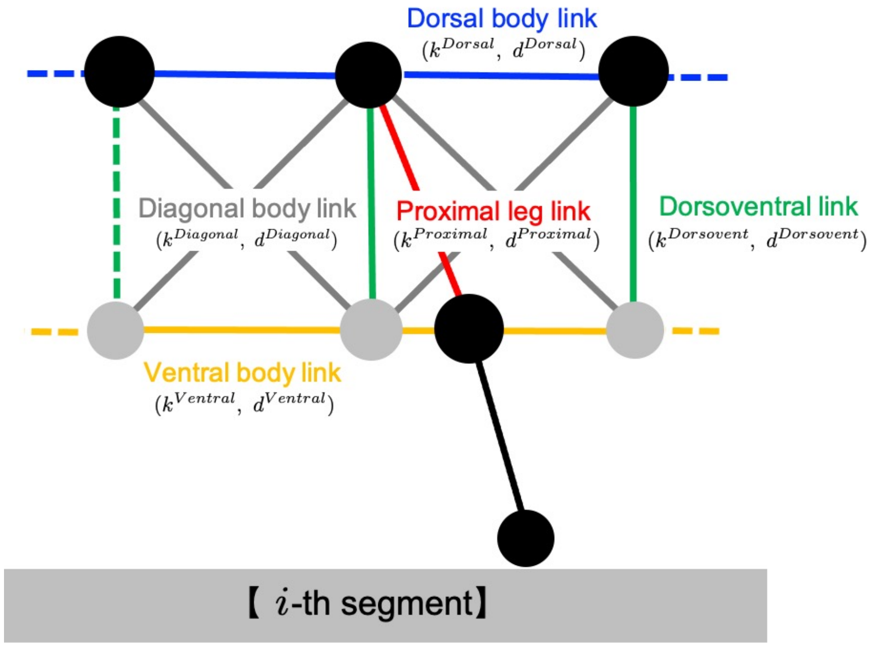

Figure S1. Definition of passive links in the mechanical system. The parameter names of the passive spring constant and damping coefficient for each link are shown in brackets.

Table S1. Body parameter values employed in the simulations.

Parameter	Value	Dimension
k^{Dorsal}	2.0×10^4	$[s^{-2}kg]$
d^{Dorsal}	2.0×10^{-1}	$[s^{-1}kg]$
$\bar{l}^{Dorsal}$	2.0×10^{-3}	[m]
$k^{Proximal}$	2.0×10^2	$[s^{-2}kg]$
$d^{Proximal}$	2.0×10^{-1}	$[s^{-1}kg]$
$\bar{l}^{Proximal}$	1.6×10^{-3}	[m]
$k^{Ventral}$	1.0×10^2	$[s^{-2}kg]$
$d^{Ventral}$	2.0×10^{-1}	$[s^{-1}kg]$
$\bar{l}^{Ventral}$	2.0×10^{-3}	[m]
$k^{Dorsovent}$	1.0×10^2	$[s^{-2}kg]$
$d^{Dorsovent}$	2.0×10^{-1}	$[s^{-1}kg]$
$\bar{l}^{Dorsovent}$	3.0×10^{-3}	[m]
$k^{Diagonal}$	2.0×10^1	$[s^{-2}kg]$
$d^{Diagonal}$	2.0×10^{-1}	$[s^{-1}kg]$
$\bar{l}^{Diagonal}$	3.6×10^{-3}	[m]

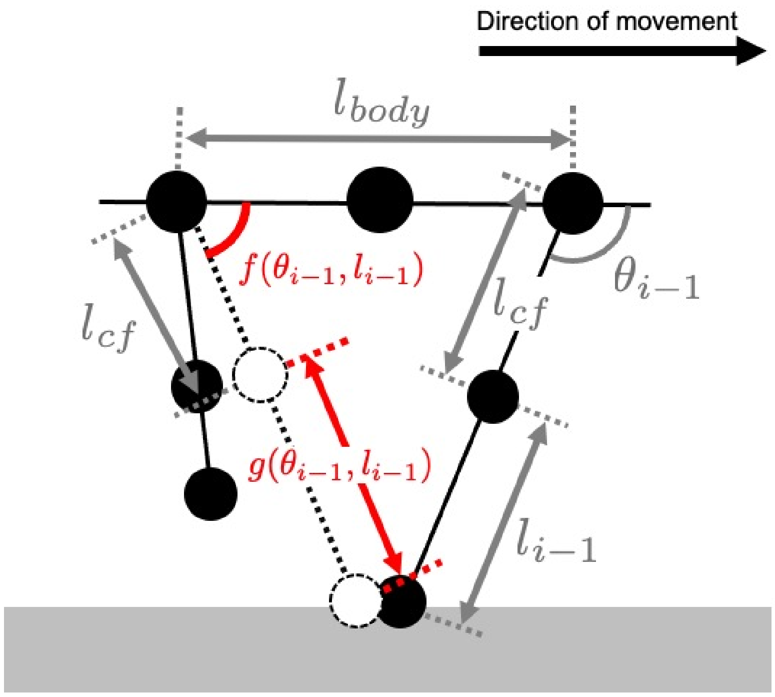

Figure S2. Definition of the parameters used for calculating inverse kinematics in Rule 3. The leg illustrated with the dashed lines denotes the target position of the i-th leg. The ventral part of the body trunk is omitted for clarity.

2 GROUND MODEL

We assumed that the ground has viscoelastic property and modeled the physical interactions between the body parts (legs and abdomen) and the ground using spring-damper system. Specifically, when the vertical positions z_i of the leg-tip and ventral body surface (*i.e.*, mass points named T_i, VB_i, VC_i in Figure 2) become lower than the ground surface ($z_i < 0$), the vertical forces from the ground (f_i^z) are applied to the mass points as follows:

$$f_i^z = \begin{cases} \max[-k^g z_i - c^g \dot{z}_i, 0] & (z_i < 0) \\ 0 & (z_i \geq 0), \end{cases} \tag{S3}$$

where k_g is the spring constant and c_g is the damping coefficient. Since it was difficult to treat static friction in simulations, we simply modeled the horizontal forces from the ground (f_i^x) using Coulomb friction:

$$f_i^x = -\mu^p f_i^z \tanh(c\dot{x}_i), \; p \in \{leg, abdomen\} \tag{S4}$$

where μ^{leg} and $\mu^{abdomen}$ are the friction coefficients for the mass points corresponding to the leg and abdomen, respectively. When $|\dot{x}_i| \gg c^{-1}$, Equation S4 describes dynamic Coulomb friction. Thus, the reaction force from the ground was described by

$$\mathbf{F}_{ground,i} = (f_i^x, f_i^z)^{\mathrm{T}} \tag{S5}$$

In the case of irregular terrain, the ground reaction forces were modeled as acting vertically and horizontally relative to the circular ground. Parameter values employed in the simulations are listed in Table S2.

Table S2. Environmental parameter values employed in the simulations.

Parameter	Value	Dimension
k^g	1.0×10^2	[kgs^{-2}]
c^g	1.0	[kgs^{-1}]
μ^{leg}	0.9	
$\mu^{abdomen}$	0.1	
c	5.0×10^2	[$\mathrm{m}^{-1}\mathrm{s}$]
